# Partial Excision and Ablative Carbon Dioxide Fractional Laser Therapy for Multiple Apocrine Hidrocystomas on the Periorbital Regions and Cheeks

**DOI:** 10.1155/2023/6318220

**Published:** 2023-07-11

**Authors:** Hiroshi Kitagawa, Isao Sugimoto, Toshinori Bito, Keiichi Yamanaka, Hiroto Terashi

**Affiliations:** ^1^Department of Dermatology, Mie University, Graduate School of Medicine, Tsu, Mie 514-8507, Japan; ^2^Department of Plastic and Aesthetic Surgery, Kobe University, Graduate School of Medicine, 7-5-2 Kusunokicho, Chuo-ku, Kobe, Hyogo 650-0017, Japan; ^3^Department of Aesthetic and Plastic Surgery, Sugimoto Aesthetic and Plastic Surgery Clinic, 5-4-10 Kotonocho, Chuo-ku, Kobe, Hyogo 651-0094, Japan; ^4^Department of Dermatology, Bito Dermatology Clinic, City-Plaza F3, 4-1-4 Asahi-Dori, Chuo-ku, Kobe, Hyogo 651-0095, Japan

## Abstract

A 51-year-old Japanese woman presented with translucent papules on the periorbital area and cheeks that had progressively enlarged over five years. She underwent a skin biopsy and was diagnosed with multiple apocrine hidrocystomas. Her lesions became more pronounced and obscured her vision when her body warmed up, such as during bathing. To alleviate her symptoms, we began treatment by partially resecting the tumors on the lower eyelids. After surgery, her vision was no longer obscured. Approximately 1.5 years later, she underwent ablative 10,600 nm carbon dioxide fractional laser therapy for the mildly enlarged apocrine hidrocystomas on her lower eyelids and cheeks. At roughly six months of follow-up, the symptoms had improved, and the cosmetic results were satisfactory, although minor scarring and hypopigmentation were still evident. These case findings underscore the effectiveness of ablative carbon dioxide fractional lasers in treating apocrine hidrocystomas.

## 1. Introduction

Apocrine hidrocystoma is a rare benign cystic tumor of the apocrine sweat glands. The lesions usually appear as solitary, well-defined, dome-shaped, translucent papules on the face, commonly around the eyes. The appearance of multiple lesions is uncommon. Histological analysis shows that these appear as unicameral or multifocal cysts in the dermis. The inner cyst wall is lined by a luminal layer of columnar cells, showing apocrine secretion, and a peripheral layer of flattened myoepithelial cells. Solitary lesions may be removed via a simple excision, electrodessication, or carbon dioxide (CO_2_) laser therapy. However, the treatment of multiple lesions is difficult because of the number and location of these lesions. Moreover, no universally accepted treatment modality is currently available for multiple lesions. In the literature, excision, topical anticholinergic agents, trichloroacetic acid, botulinum toxin, CO_2_ laser, and 1450 nm diode laser have been suggested as therapeutic options for multiple lesions. Here, we present an interesting case of a patient with multiple apocrine hidrocystomas on the periorbital regions and cheeks, which functionally and cosmetically improved after partial resection and therapy using an ablative CO_2_ fractional laser system (CO_2_ FS).

## 2. Case Presentation

**Figure 1:**
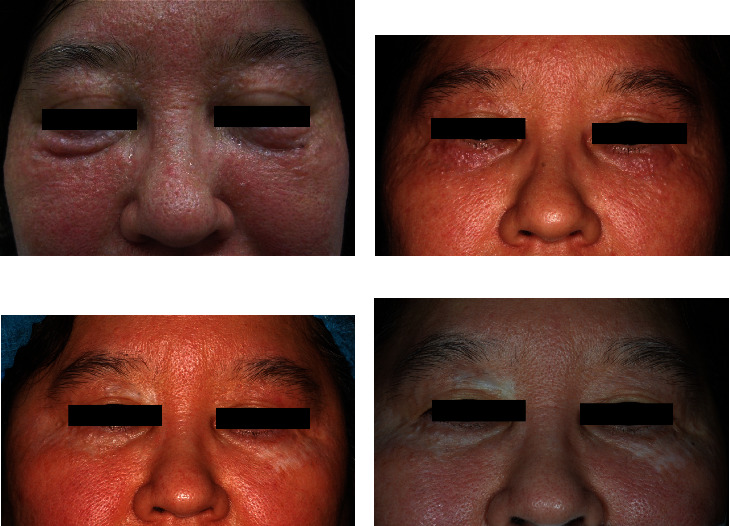
(a) Multiple skin-colored translucent papules on the periorbital regions and cheeks. (b) Nine months after surgical therapy, the lesions on the lower eyelids do not obscure her vision. (c) The lesions have improved six months after laser therapy, even though partial scarring and hypopigmentation remain. (d) The persistence of the effect for CO_2_ FS was considered to be constant for at least three years.

**Figure 2:**
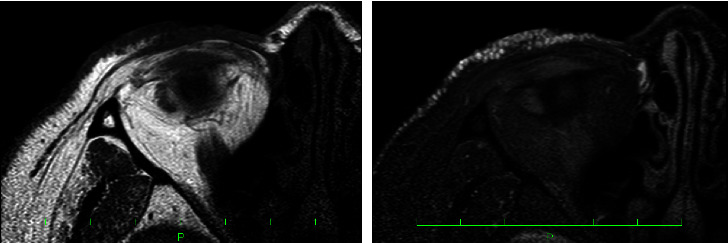
Magnetic resonance image of the face shows 1 to 2 mm papules within the dermis, which exhibit a low intensity on T1-weighted imaging and a high intensity on T2-weighted imaging.

**Figure 3:**
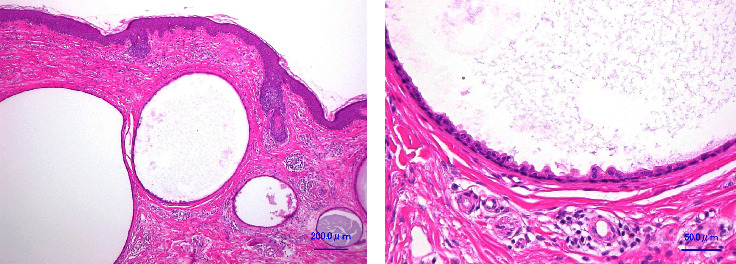
Unilocular cystic structure within the dermis on staining with hematoxylin-eosin. The lining of the cysts shows an inner layer of apocrine cells and an outer layer of myoepithelial cells. (a) ×40 magnification. (b) ×100 magnification.

A 51-year-old Japanese woman presented with translucent papules on the periorbital area and cheeks that had gradually enlarged over five years. When her body was warm, such as during bathing, the lesions became more pronounced and obscured her vision. She underwent a skin biopsy at a local clinic and was diagnosed with multiple apocrine hidrocystomas. A physical examination revealed numerous dome-shaped, skin-colored, translucent lesions up to 2 mm in diameter on the periorbital regions and both cheeks (Figure 1(a)). Magnetic resonance imaging revealed 1 to 2 mm papules within the dermis, which exhibited a low and high intensity on T1- and T2-weighted imaging, respectively (Figure 2). Her family history was unremarkable. We started treatment with a partial resection of the lower eyelids. Histopathological analysis revealed multiple dermal cysts. The inner layer of the cysts was lined by a row of columnar secretory cells peripheral to the layer lined by flattened myoepithelial cells (Figure 3). After surgery, the patient's vision was no longer obscured (Figure 1(b)). At 1.5 years of follow-up, an ablative 10,600 nm CO_2_ FS (UltraPulse® Deep FX™; Lumenis Inc., Yokneam, Israel) was used to treat slightly enlarged apocrine hidrocystomas on her lower eyelids and cheeks. In the treatment area, local anesthesia was performed using 1% lidocaine with epinephrine and eye shields were used for eye protection. The lesions were treated with one pass of the laser at 30–50 mJ and at 5–25% density. In her lower eyelids, the upper limits of 40 mJ and 20% density were set for eye protection [1]. At approximately six months and three years of follow-up, the symptoms showed continued improvement, and the cosmetic results were satisfactory, although some minor scarring and hypopigmentation persisted (Figures 1(c) and 1(d)).

## 3. Discussion

Hidrocystomas are benign nevoid tumors of the skin that appear mainly on the face [2]. Anzai et al. analyzed 167 Japanese cases of apocrine hidrocystomas and found no association between their occurrence and the patients' sex or age. In 123 cases (73.7%), the tumor was found on the face or scalp, especially in the periorbital area, but multiple hidrocystomas were rare [3]. Multiple apocrine hidrocystomas may be a symptom of Schopf−Schulz−Passarge syndrome, a rare autosomal recessive syndrome involving ectodermal dysplasia [4].

No fixed treatment strategy is currently available for multiple apocrine hidrocystomas. However, 35 patients with periorbital syringomas were treated effectively using a CO_2_ FS [5]. Laser fluences were delivered to the entire lower eyelids at a pulse energy of 100 mJ and at a density of 100 spots/cm^2^ (coverage area of 8.6%). The histological analyses of 11 pretreatment specimens revealed that the mean depth of the syringomas was 1.06 ± 0.34 mm. The mean maximal depth of the necrotic columns was 1236.3 *μ*m at a pulse energy of 100 mJ. In the present study, the mean depth of the apocrine hidrocystomas was 0.72 ± 0.33 mm (range, 0.27–1.45 mm). The CO_2_ FS showed a correlation between energy and penetration depth. In another report, energy settings of 5–20 mJ with a CO_2_ FS resulted in tissue injury up to depths of 0.5–2 mm [6]. We treated multiple lesions with energy settings of 30–50 mJ and 5–25% density. Although we did not perform a histological analysis after CO_2_ FS therapy, we thought that the energy was sufficient to reduce multiple lesions.

We achieved satisfactory symptomatic and cosmetic results using the CO_2_ FS although partial mild scarring and hypopigmentation remained. Complications of CO_2_ FS therapy include the risk of scarring, scar contracture, and depigmentation, particularly in the eyelid, which may impair the ability to open and close the eyelid due to lagophthalmos and contracture. Another significant concern with CO_2_ FS is that while repeated treatments can reduce the risk of recurrence, they simultaneously increase the risk of complications, thus limiting the extent of its use. In our case, three years post CO_2_ FS treatment, some recurrence necessitated additional CO_2_ FS treatments. Although the treated lesions have not fully reverted to their original state, making it challenging to evaluate the overall effect, we observed a reduction in tumor enlargement. Furthermore, we believe that the treatment effect was consistently sustained for at least three years. No other reports have documented using a CO_2_ FS to treat multiple apocrine hidrocystomas. Therefore, the current case findings highlight the utility of this system as a therapeutic option for this rare condition. Nevertheless, additional studies are warranted to establish an optimal treatment for obtaining more desirable clinical outcomes.

## Data Availability

The data used to support the findings of the study are included within the article.

## Abbreviations

CO_2_: Carbon dioxide
CO_2_ FS: An ablative CO_2_ fractional laser system.
